# Association between oxidative balance score in adults with and without chronic kidney disease: 2011–2028 NHANES

**DOI:** 10.3389/fnut.2024.1374719

**Published:** 2024-04-23

**Authors:** Yuyu Cao, Yishan Zhou, Yanghong Zhong, Xianyong Liao, Xushan Chen, Ying Pi

**Affiliations:** Seventh Clinical Medical College, Guangzhou University of Chinese Medicine, Shenzhen, China

**Keywords:** NHANES, oxidative balance score, chronic kidney disease, dietary OBS, lifestyle OBS

## Abstract

**Introduction:**

Oxidative stress status is associated with CKD; however, few studies have investigated this association. The oxidative balance score (OBS) reflects systemic stress status and consists of 16 anti-and pro-oxidant dietary factors and four anti-and pro-oxidant lifestyle factors. Higher OBS implies exposure to more antioxidants. The purpose of this study was to explore the association between OBS and CKD.

**Methods:**

We enrolled 8,134 study participants from the 2011–2018 National Health and Nutrition Examination Survey and obtained OBS by adding the 20 dietary and lifestyle factors. Based on OBS, the participants were divided into three groups. We performed logistic regression, subgroup analyzes, and restricted cubic spline regression to explore the association between OBS and CKD. In addition, we tested the adjusted model.

**Results:**

OBS was negatively associated with CKD (OR: 0.54; 0.66, 0.82). After adjusting for all confounders, when dietary OBS was >20, the prevalence of CKD was reduced by 42% for each unit increase in OBS (*p* < 0.05). The negative associations of total OBS, dietary OBS, and lifestyle OBS with CKD were more significant in the female group. When the total OBS was ~20, the trend of decreasing prevalence in the female group was more significant.

**Conclusion:**

OBS is negatively associated with chronic kidney disease.

## 1 Introduction

Chronic kidney disease (CKD) is a silent killer and its prevalence is increasing each year. It is expected that CKD will become the fifth leading cause of death worldwide by 2040. When treating CKD, the goal is to prevent or delay the progressive decline in renal function. CKD poses a considerable financial burden due to the cost of diagnoses and treatment as well as the later stage of renal replacement therapy (1–3).One survey showed that the prevalence of CKD stages 3–5 was 6.7% among Americans 16 years and over. The risk factors for CKD include age, smoking, alcohol consumption, diabetes mellitus, and hypertension. Therefore, it is imperative that we focus on preventive and curative approaches of CKD (2, 4).

Oxidative stress, which results from an imbalance between oxidant and antioxidants, disrupts cellular structure and function, contributes to organ tissue damage, and leads to organ degenerative changes (5). Reactive oxygen species play an important role in redox homeostasis, and reactive oxygen species production is closely linked to mitochondria (6). The kidneys are rich in mitochondria and, consequently, more susceptible to oxidative stress (7).

CKD is strongly associated with oxidative stress, a risk factor for various diseases. High levels of oxidative stress are present in the early stages of CKD, and this is evident from the redox state imbalance characteristic in the etiology of polynephropathy (8–10). Epidemiological studies have shown the health-promoting and disease-preventing effects of fruits and vegetables, which are rich in antioxidants. However, fruits and vegetables are rich in potassium, the intake of which should be minimized by CKD patients. Hence, we need to promote the intake of antioxidants to minimize oxidative stress (11, 12) and prevent CKD (13–15).

Even though the oxidant/antioxidant balance is largely dependent on endogenous enzymes, modifiable factors such as diet, drugs, and lifestyle do affect it to some extent. Various studies have shown that levels of pro-oxidant biomarkers increase and levels of antioxidant biomarkers decrease in CKD patients (16–19). However, the use of pro-or antioxidant biomarkers to measure oxidative stress has its limitations. Pro-or antioxidant biomarkers are obtained from the binding of reactive oxygen species to specific biomolecules; however, the technical standards required for this marker are too high, storage requirements are high, and it must not be confused with food, which is a major limitation. Therefore, we used OBS to assess the oxidative stress status of the organism (20).

Van Hoydonck et al. (21) developed an oxidative balance score (OBS), and Son et al. (22) found that healthy diets and lifestyles that increase OBS may be beneficial in the prevention of CKD among East Asian adults. Notably, OBS represents the “external oxidative stress” to which an individual is exposed and not “the oxidative milieu of the organism” in the setting of CKD. The objective of our study was to explore the association between OBS and CKD in US adults.

## 2 Materials and methods

### 2.1 Data source and study participants

**Figure 1:**
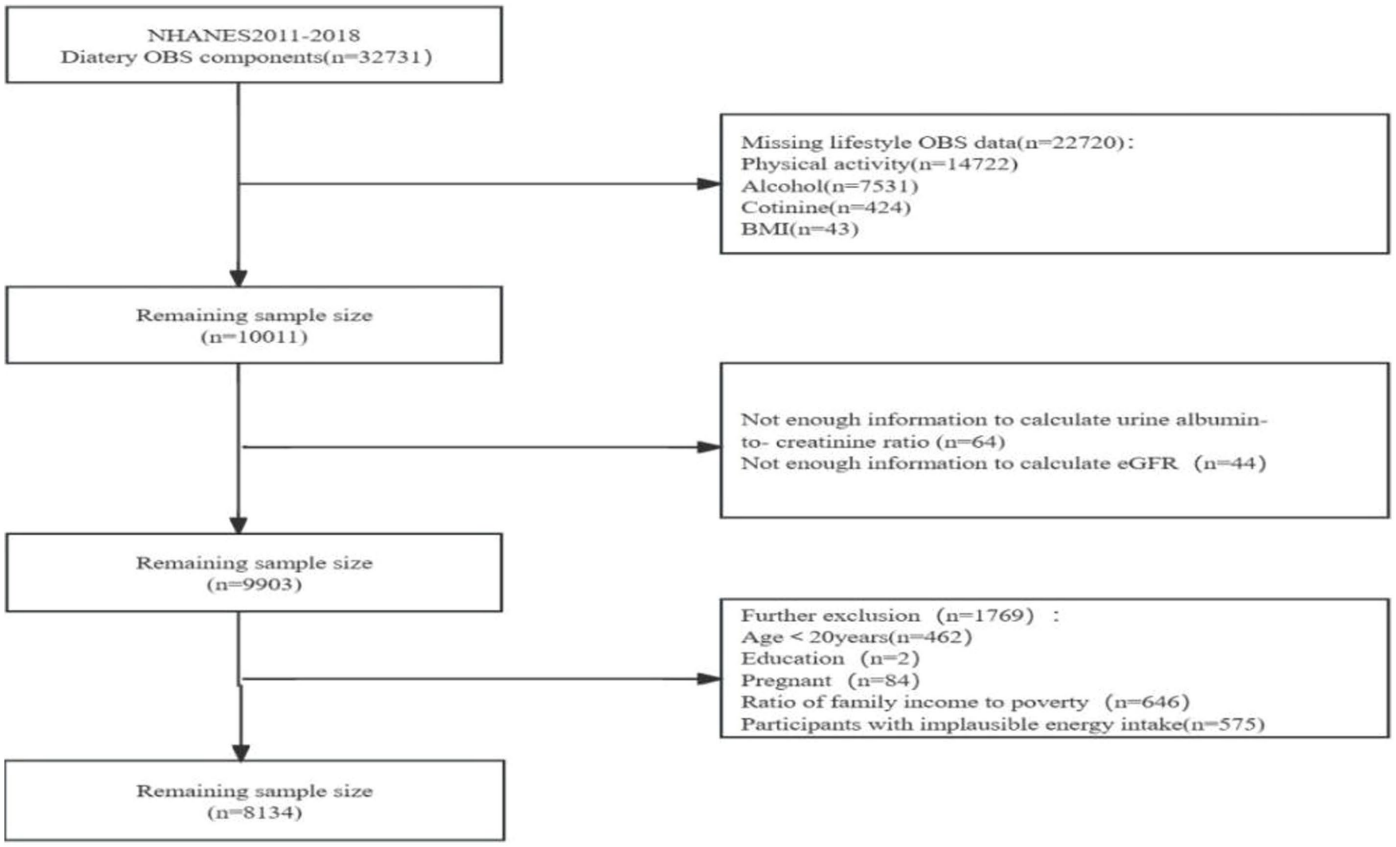
Flowchart of the selection of study participants.

We obtained the data from the National Health and Nutrition Examination Survey (NHANES), a project designed to evaluate the health and nutritional status of populations in the US. We obtained approval from the Ethics Review Board of the U.S. National Center for Health Statistics, and all participants signed an informed consent. We recruited 32,731 participants with dietary OBS from NHANES surveys: 2011–2012, 2013–2014, 2015–2016, and 2017–18. We excluded 22,720 participants who had no information on lifestyle OBS, including 14,722 participants who had no information on physical activity, 7,531 participants who had no information on alcohol consumption, 424 participants who had no information on smoking frequency, and 43 participants who had no information on body mass index (BMI). Additionally, we ruled out 108 participants who had no information on estimated glomerular filtration rate (eGFR) or albumin-to-creatinine ratio (4) We excluded 646 participants who had no information on income-to-poverty ratio (IPR), two participants who had no information on education level, 575 participants with implausible energy intakes (i.e., outside the range of 800–4,200 kcal/d for males and 500–3,500 kcal/d for females) (23, 24), those <20 y of age, and 84 pregnant women. Ultimately, we enrolled 8,134 participants in the study (Figure 1).

### 2.2 OBS definitions

OBS was calculated as previously reported (21). OBS consists of 16 dietary OBS (14 antioxidants and two pro-oxidants) and four lifestyle OBS (one antioxidant and three pro-oxidants). Total OBS is the sum of the scores of these 20 components. High OBS is indicative of a higher exposure or consumption of antioxidants. Lifestyle incorporates four indicators: alcohol consumption, smoking frequency/status, BMI, and physical activity. Alcohol consumption was defined as the average daily amount of alcohol consumed in the past year. Smoking frequency/status was assessed by measuring plasma cotinine levels, the main metabolite of nicotine, which has a longer half-life in the blood than nicotine and an indicator of “active” or “passive” smoking. BMI was calculated as weight (kg) divided by squared height (m). Dietary incorporates sixteen indicators such as dietary fiber, carotenoids, riboflavin, niacin, vitamin B6, total folate, vitamin B12, vitamin C, vitamin E, calcium, magnesium, zinc, copper, selenium, total fat, and iron were obtained from a 24-h dietary recall interview (24HR) in the Mobile Examination Center (MEC).

The physical activity data included work-related activities of high and moderate intensity as well as physical activity during leisure time in three categories: 1) traveling on foot or by bicycle, 2) high, and 3) moderate intensity leisure time activities. The data were obtained from the physical activity questionnaire in NHANES. The assessment of physical activity was calculated from metabolic equivalent score × weekly frequency of each physical activity × duration of each physical activity (25).

OBS tertiles were calculated, and all components were classified into three groups. Each components were assigned scores ranging from 0 to 2,with more exposure to antioxidants resulting in higher scores, and the opposite for pro-oxidants (Table 1).

**Table 1:** Components of the oxidative balance score.

OBS components	Property	Males			Females		
		0	1	2	0	1	2
*Dietary OBS components*							
Dietary fiber (g/d)	A	<12.40	12.40–20.70	≥20.70	<10.50	10.50–17.30	≥17.30
Carotene (RE/d)	A	<69.12	69.12–238.56	≥238.56	<69.29	69.29–299.20	≥299.20
Riboflavin (mg/d)	A	<1.66	1.66–2.52	≥2.52	<1.33	1.33–1.99	≥1.99
Niacin (mg/d)	A	<22.19	22.19–33.18	≥33.18	<16.07	16.07–23.81	≥23.81
Vitamin B6 (mg/d)	A	<1.67	1.67–2.58	≥2.58	<1.22	1.22–1.94	≥1.94
Total folate (mcg/d)	A	<306.00	306.00–487.00	≥487.00	<240.00	240.00–376.92	≥376.92
Vitamin B12 (mcg/d)	A	<3.15	3.15–6.60	≥6.60	<2.19	2.19–4.59	≥4.59
Vitamin C (mg/d)	A	<28.70	28.70–91.68	≥91.68	<28.40	28.40–82.88	≥82.88
Vitamin E (ATE; mg/d)	A	<6.19	6.19–10.50	≥10.50	<5.42	5.42–9.14	≥9.14
Calcium (mg/d)	A	<689.00	689.00–1144.79	≥1144.79	<594.00	594.00–947.00	≥947.00
Magnesium (mg/d)	A	<257.00	257.00–372.00	≥372.00	<210.00	210.00–301.00	≥301.00
Zinc (mg/d)	A	<9.11	9.11–13.99	≥13.99	<6.72	6.72–10.30	≥10.30
Copper (mg/d)	A	<0.98	0.98–1.43	≥1.43	<0.83	0.83–1.23	≥1.23
Selenium (mcg/d)	A	<100.10	100.10–146.70	≥146.70	<73.20	73.20–109.30	≥109.30
Total fat (g/d)	P	≥103.46	68.62–103.46	<68.62	≥83.11	54.60–83.11	<54.60
Iron (mg/d)	P	≥17.30	11.54–17.30	<11.54	≥13.40	8.82–13.40	<8.82
*Lifestyle OBS components*							
Physical-activity (MET-minute/week)	A	<1680.00	1680.00–5760.00	≥5760.00	<1020.00	1020.00–3298.42	≥3298.42
Alcohol consumption (g/d)	P	≥3.00	2.00–3.00	<2.00	≥2.00	1.00–2.00	<1.00
Body mass index (kg/m^2^)	P	≥30.10	25.70–30.10	<25.70	≥31.50	25.00–31.50	<25.00
Cotinine (ng/mL)	P	≥4.17	0.02–4.17	<0.02	≥0.11	0.01–0.11	<0.01

OBS, oxidative balance score; A, antioxidant; P, pro-oxidant; RE, retinal equivalent; ATE, alpha-tocopherol equivalents; MET, metabolic equivalent.

### 2.3 CKD definition

According to the purpose of this study, the included participants were divided into those with and without CKD.CKD was defined by eGFR <60 mL/min/1.73 m^2^ and/or albuminuria (urinary albumin/creatinine ratio > 30 mg/g) (26). Based on Kidney Disease: Improving Global Outcomes (KDIGO), individuals with eGFR <60 mL/min/1.73 m^2^ or albuminuria (urinary albumin/creatinine ratio > 30 mg/g were included the study) (27–29).

### 2.4 Confounders

We considered the following variables as confounders: age, race/ethnicity (non-Hispanic white, non-Hispanic black, Mexican American, and other races/ethnicities), gender, education level (< high school, high school, > high school), IPR, caffeine intake, energy intake, history of hypertension, and history of diabetes.

### 2.5 Statistical analyzes

A normality test on the continuous variables revealed that the continuous variables did not follow a normal distribution. For categorical and non-normal continuous variables, we used Chi-squared test and Kruskal–Wallis test, respectively, to correctly assess the differences in the characteristics of the variables in the different OBS groups (tertile). We used medians (IQR) for non-normal continuous variables and numbers (percentages, %) for categorical variables.

A logistic regression model was used to analyze the association between OBS and CKD. In the crude model, we made no adjustments for potential confounding factors. Model 1 adjusted for age and gender; model 2 adjusted for age, gender, race/ethnicity, education level, and IPR; and model 3 adjusted for age, gender, race/ethnicity, education level, IPR, caffeine intake, and energy intake. Additionally, we assessed any potential nonlinear associations between OBS and CKD using restricted cubic spline regression.

All statistical analyzes were performed using IBM SPSS Statistics 26.0 and Free Statistics. A two-sided *p* < 0.05 was considered statistically significant.

## 3 Results

### 3.1 Baseline characteristics

We enrolled 8,134 participants: 4,372 males and 3,762 females. Table 2 shows the baseline characteristics of the participants. The number of participants with/without CKD was 1197/6937, with a prevalence of 14.7%. Meanwhile, the number of participants with CKD and co-morbidities (e.g., diabetes mellitus and hypertension) decreased with higher OBS. Also, UACR decreased with higher OBS. There were more males and females in the highest OBS tertile than in the lowest OBS tertile. Compared to the lowest OBS tertile, the highest OBS tertile had higher IPR, greater education level, and higher caffeine and energy intakes and were mainly non-Hispanic white. The difference in age and eGFR among the three OBS tertiles was not statistically significant.

**Table 2:** Baseline characteristics of overall study participants based on the oxidative balance score tertile.

	ALL	T1 (< 16)	T2 (16–23)	T3 (≥ 23)	*p* value
	*N* = 8,134	*N* = 2,557	*N* = 2,590	*N* = 2,987	
*Age*	44 (31, 59)	43 (31, 59)	45 (31, 60)	44 (31, 58)	0.191
*Gender, n (%)*					< 0.001
Males	4,372 (53.7)	1,298 (50.8)	1,415 (54.6)	1,659 (55.5)	
Females	3,762 (46.3)	1,259 (49.2)	1,175 (45.4)	1,328 (44.5)	
*Race/ethnicity, n (%)*					< 0.001
Mexican American	997 (12.3)	294 (11.5)	311 (12)	392 (13.1)	
Other Hispanic	724 (8.9)	229 (9)	231 (8.9)	264 (8.8)	
Non-Hispanic White	3,558 (43.7)	1,014 (39.7)	1,141 (44.1)	1,403 (47)	
Non-Hispanic Black	1,642 (20.2)	704 (27.5)	513 (19.8)	425 (14.2)	
Other races, including multi-racial	1,213 (14.9)	316 (12.4)	394 (15.2)	503 (16.8)	
*Ratio of family income to poverty*	2.6 (1.3, 4.8)	2.0 (1.0, 3.8)	2.6 (1.3, 4.5)	3.2 (1.6, 5.0)	< 0.001
*Education level, n (%)*					< 0.001
< High school	1,095 (13.5)	441 (17.2)	359 (13.9)	295 (9.9)	
High school/general educational development	1,697 (20.9)	655 (25.6)	564 (21.8)	478 (16)	
> High school	5,342 (65.7)	1,461 (57.1)	1,667 (64.4)	2,214 (74.1)	
*Co-morbidities, yes (%)*					
Diabetes history	768 (9.4)	267 (10.4)	262 (10.1)	239 (8)	0.003
Hypertension history	2,520 (31.0)	874 (34.2)	817 (31.5)	829 (27.8)	< 0.001
*Energy (kcal)*	2042 (1,542, 2,631)	1,524 (1,193, 1945)	2027 (1,628, 2,515)	2,547 (2059, 3,089)	< 0.001
*Caffeine (mg)*	106 (22, 216)	93 (14, 193)	108 (22, 218)	122 (29, 236)	< 0.001
*eGFR(mL/min/1.73 m^2^)*	86.4 (67.0, 105.4)	85.5 (65.9, 105.8)	86.3 (66.8, 105.5)	87.0 (68.4, 105.0)	0.205
*UACR(mg/g)*	6.6 (4.4, 11.8)	7.2 (4.7, 13.5)	6.7 (4.4, 12.0)	6.2 (4.2, 10.5)	< 0.001
*Chronic kidney disease, n (%)*					0.004
No	6,937 (85.3)	2,144 (83.8)	2,196 (84.8)	2,597 (86.9)	
Yes	1,197(14.7)	413 (16.2)	394 (15.2)	390 (13.1)	

eGFR, Estimated glomerular filtration rate; UACR, Urinary albumin/urine creatinine ratio.

### 3.2 Association between total OBS and CKD

Logistic regression analyzes revealed the association between OBS and CKD (Table 3). In model 4, which adjusted for all potential confounders, compared to the lowest OBS tertile, the highest OBS tertile had a more significant negative association with CKD (T2: OR = 0.72 [0.59, 0.87], *p* = 0.001; T3: OR = 0.54 [0.66, 0.82], *p* < 0.001). All remained relatively stable across models and were statistically significant.

**Table 3:** Multivariable-adjusted odds ratio (95% confidence intervals) of chronic kidney disease (CKD) by oxidative balance score’s tertile.

	T1(<16)	T2 (16–23)	*p* value	T3 (≥23)	*p* value
CKD/Non-CKD	413/2144	394/2196		390/2597	
Model 1	1 (Ref)	0.79 (0.66 ~ 94)	0.009	0.65 (0.55 ~ 0.78)	<0.001
Model 2	1 (Ref)	0.78 (0.65 ~ 0.93)	0.007	0.63 (0.52 ~ 0.76)	<0.001
Model 3	1 (Ref)	0.71 (0.59 ~ 0.86)	0.001	0.52 (0.42 ~ 0.66)	<0.001
Model 4	1 (Ref)	0.72 (0.59 ~ 0.87)	0.001	0.54 (0.66 ~ 0.82)	<0.001

Model 1: adjusted for age and gender.

Model 2: adjusted for age, gender, race/ethnicity, education level, and income-to-poverty ratio.

Model 3: adjusted for age, gender, race/ethnicity, education level, income-to-poverty ratio, caffeine intake, and energy intake.

Model 4: adjusted for age, gender, race/ethnicity, education level, income-to-poverty ratio, caffeine intake, energy intake, diabetes mellitus history, and hypertension history.

### 3.3 Association between dietary and lifestyle OBS and CKD

Table 4 shows the multivariate logistic regression analysis of the association between different OBS and CKD, including dietary and lifestyle OBS. After adjusting for all potential confounders, we found a significant association between CKD and dietary OBS (OR = 0.58 [0.46, 0.73], *p* < 0.001). In other words, when dietary OBS was greater than 20, each unit increase in dietary OBS was associated with a 42% decrease in the number of participants with CKD. Higher lifestyle OBS was negatively associated with risk of CKD after adjusting for all potential confounders (OR = 0.76 [0.63, 0.91], *p* = 0.003). The results of the trend test indicated that the decrease observed was statistically significant (*p* < 0.05). Additionally, there was an interaction between dietary OBS and lifestyle OBS among the participants (*p* < 0.001) (2).

**Table 4:** OR estimates for associations between dietary/lifestyle OBS and chronic kidney disease (CKD).

OBS	T1	T2(OR 95%CI)	*p* value	T3(OR 95%CI)	*P* value	*p* for trend
Dietary OBS						
Crude model	1 (Ref)	0.82 (0.7 ~ 0.95)	0.008	0.72 (0.62 ~ 0.83)	<0.001	<0.001
Model 1	1 (Ref)	0.75 (0.63 ~ 0.89)	0.001	0.69 (0.58 ~ 0.83)	<0.001	<0.001
Model 2	1 (Ref)	0.79 (0.66 ~ 0.96)	0.014	0.78 (0.65 ~ 0.94)	0.008	0.007
Model 3	1 (Ref)	0.75 (0.62 ~ 0.92)	0.005	0.70 (0.55 ~ 0.88)	0.003	0.002
Model 4	1 (Ref)	0.69 (0.57 ~ 0.84)	<0.001	0.58 (0.46 ~ 0.73)	<0.001	<0.001
Life OBS						
Crude model	1 (Ref)	1.03 (0.86 ~ 1.23)	0.751	1.32 (1.14 ~ 1.53)	<0.001	<0.001
Model 1	1 (Ref)	0.9 0 (0.73 ~ 1.11)	0.317	0.74 (0.62 ~ 0.89)	0.001	0.001
Model 2	1 (Ref)	0.95 (0.76 ~ 1.17)	0.609	0.81 (0.67 ~ 0.98)	0.031	0.024
Model 3	1 (Ref)	0.95 (0.76 ~ 1.17)	0.614	0.81 (0.67 ~ 0.98)	0.033	0.026
Model 4	1 (Ref)	0.92 (0.74 ~ 1.13)	0.425	0.76 (0.63 ~ 0.91)	0.003	0.002
Dietary OBS *Lifestyle OBS p for interaction^2^						< 0.001

*Crude model: unadjusted model.

Model 1: adjusted for age and gender.

Model 2: adjusted for age, gender, race/ethnicity, education level, and income-to-poverty ratio.

Model 3: adjusted for age, gender, race/ethnicity, education level, income-to-poverty ratio, caffeine intake, and energy intake.

Model 4: adjusted for age, gender, race/ethnicity, education level, income-to-poverty ratio, caffeine intake, energy intake, diabetes mellitus history, and hypertension history.

The specific range for the tertile: dietary OBS: T1 [2–13]; T2 [13–20]; T3 [20–31]; Lifestyle OBS: T1 [0–3]; T2 [3–4]; T3 [4–8].

### 3.4 Subgroup analyzes

**Figure 2:**
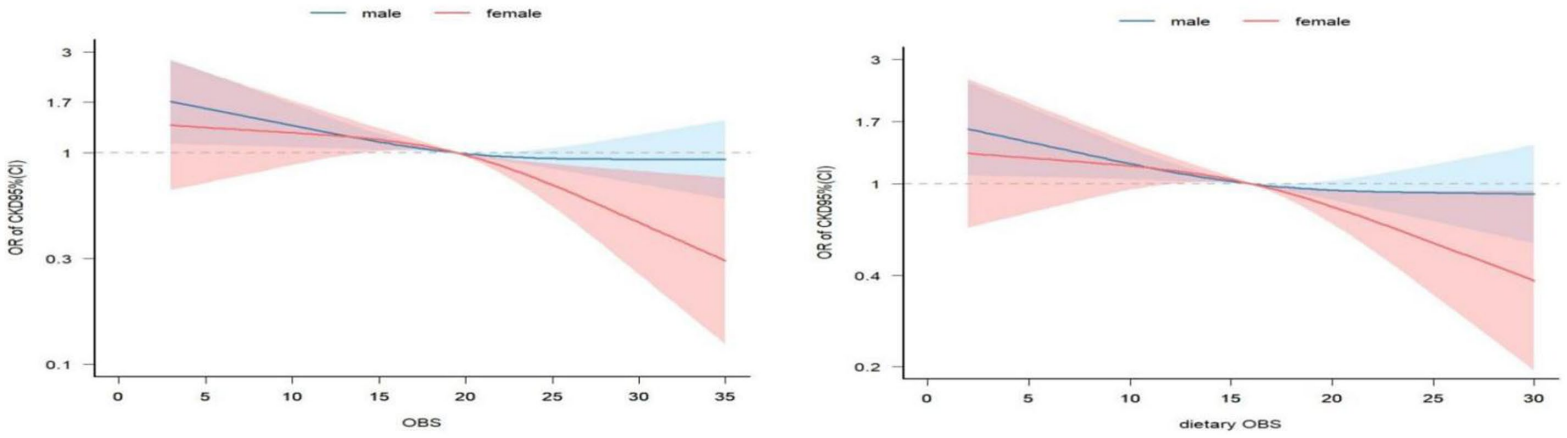
Analysis of restricted cubic spline regression. Adjusted restricted cubic spline models were adjusted for age, gender, race/ethnicity, poverty-to-income ratio, caffeine intake, energy intake, diabetes mellitus history, and hypertension history.

Table 5 shows the association between total OBS, dietary OBS, and lifestyle OBS and CKD for males and females based on multivariate logistic regression. After adjusting for all potential confounders, we found that the association between different OBS and CKD was negatively more significant for females than for males. Interestingly, when analyzing the interaction between gender and different OBS, in the model that adjusted for all potential confounders (model 4), the only interaction with gender was lifestyle OBS (*p* < 0.05). There was no interaction between total OBS, dietary OBS, and gender (*p* > 0.05). The results were consistent in RCS (Figure 2), in which we could visualize the association between total OBS, dietary OBS and CKD. The association between lifestyle OBS and CKD differed by gender. When the total OBS was >20, the decline in CKD prevalence was more significant among females. The specific range of values was consistent with the results presented in Tables 2, 4.

**Table 5:** OR estimates for the association between different OBS and chronic kidney disease (CKD) in males and females.

	T1	T2 (OR, 95% CI)	*p* value	T3 (OR, 95% CI)	*p* value	*p* for trend	*p* for effect modification
OBS							
Adjusted							0.131
Male	1 (Ref)	0.81 (0.65 ~ 1.02)	0.075	0.74 (0.57 ~ 0.96)	0.025	0.027	
Female	1 (Ref)	0.66 (0.44 ~ 0.99)	0.047	0.453 (0.26 ~ 0.71)	0.001	0.001	
Dietary OBS							
Adjusted							0.423
Male	1 (Ref)	0.75 (0.6 ~ 0.94)	0.011	0.75 (0.57 ~ 0.97)	0.031	0.03	
Female	1 (Ref)	0.76 (0.5 ~ 1.14)	0.183	0.54 (0.33 ~ 0.89)	0.015	0.015	
Lifestyle OBS							
Adjusted							0.045
Male	1 (Ref)	1.02 (0.79 ~ 1.33)	0.861	0.95 (0.76 ~ 1.19)	0.666	0.604	
Female	1 (Ref)	0.86 (0.57 ~ 1.29)	0.463	0.63 (0.42 ~ 0.94)	0.023	0.023	

*Adjusted Model: adjusted for age, gender, race/ethnicity, income-to-poverty ratio, caffeine intake, energy intake, diabetes mellitus history, and hypertension history.

## 4 Discussion

Our study explored the association between OBS and with/without CKD. After adjusting for all confounders, total, dietary, and lifestyle OBS were negatively associated with CKD. These results underscore the connection between antioxidants acquired through dietary patterns and lifestyle choices and their association with protection against CKD.

Our findings are consistent with previous epidemiological studies that showed that oxidative stress is negatively associated with CKD, i.e., higher OBS is associated with a lower prevalence of CKD (22, 30). Several study findings have shown that renal insufficiency is associated with oxidative stress and that oxidative stress markers are elevated in patients with impaired renal function (31–33). Oxidative stress markers such as plasma malondialdehyde and oxidized low-density lipoprotein increase with the pathogenesis of CKD (34). Our study used several markers to assess oxidative stress in CKD participants. Studies have found no single marker of oxidative stress levels, and even though the redox state of serum albumin may reflect the degree of oxidative stress, its measurement is complex and not commonly used in clinical practice (35, 36).

Our study had several strengths. First, our study incorporated both diet and lifestyle to assess oxidative stress, without resorting to samples such as blood or urine, making it more economical, convenient, and readily available. Second, our study was stratified according to gender to make the results more relevant, and the results showed that dietary antioxidants were more protective for females, with a smaller OR for females than for males in the different strata. In other words, dietary changes may have a greater benefit in reducing the prevalence of CKD in females. Oxidative stress is physiologically different between males and females. For example, estrogen prevents the deterioration of kidney function through its antioxidant and anti-inflammatory properties (37). There is evidence that renal function declines faster in males than in females, which may be due to unhealthy lifestyles in males and to the deleterious role that androgens play in oxidative stress, activating the renin-angiotensin system and worsening fibrosis in damaged kidneys (38, 39). Estrogen protects rats from oxidative stress by inducing the expression of antioxidant genes (40).

OBS is not only relevant to CKD, but also as a part of health education to prevent the disease.

Nevertheless, this study had some limitations. First, considering that all OBS components were used with the same weight, OBS might not sufficiently reflect the actual biological contribution. However, studies focused on the association between OBS and the risk of colorectal adenoma and prostate cancer revealed no significant differences in the results from weighted and unweighted OBS (41, 42). Second, due to database limitations, it was difficult to incorporate all oxidative stress related diet and lifestyle in the OBS. Third, we did not follow up with the study participants to assess the association between OBS and CKD outcomes or end-stage renal disease outcomes. Finally, because our study had a cross-sectional design, we could not establish a causal relationship between OBS and CKD; therefore, more prospectively designed studies are needed to demonstrate the effectiveness of OBS. Nevertheless, our study findings, which revealed a negative association between OBS and CKD, have clinical relevance.

## 5 Conclusion

After adjusting for potential confounders, we found that OBS was negatively associated with CKD. The association between OBS and CKD warrants further research.

## Scope statement

This cross-sectional study explored the relationship between OBS and CKD in US adults using the NHANES database. OBS consisted of both dietary and lifestyle components. Our target modules are Clinical Nutrition.

## Data availability statement

The original contributions presented in the study are included in the article/supplementary material, further inquiries can be directed to the corresponding authors.

## Ethics statement

Ethical approval was not required for the study involving humans in accordance with the local legislation and institutional requirements. Written informed consent to participate in this study was not required from the participants or the participants' legal guardians/next of kin in accordance with the national legislation and the institutional requirements.

## Author contributions

YC: Conceptualization, Data curation, Investigation, Methodology, Software, Writing -original draft. YiZ: Conceptualization, Data curation, Investigation, Methodology, Software, Writing – original draft. YaZ: Writing – original draft. XL: Writing – review and editing. XC: Conceptualization, Writing – review and editing. YP: Conceptualization, Writing – review and editing.

## References

[1] Bikbov B Purcell CA Levey AS Smith M Abdoli A Abebe M. Global, regional, and national burden of chronic kidney disease, 1990–2017: a systematic analysis for the global burden of disease study 2017. Lancet. (2020) 395:709–33. doi: 10.1016/S0140-6736(20)30045-3, PMID: 32061315 PMC7049905

[2] Foreman KJ Marquez N Dolgert A Fukutaki K Fullman N McGaughey M. Forecasting life expectancy, years of life lost, and all-cause and cause-specific mortality for 250 causes of death: reference and alternative scenarios for 2016–40 for 195 countries and territories. Lancet. (2018) 392:2052–90. doi: 10.1016/S0140-6736(18)31694-5, PMID: 30340847 PMC6227505

[3] Ortiz A Asociación Información Enfermedades Renales Genéticas (AIRG-E), European Kidney Patients' Federation (EKPF), Federación Nacional de Asociaciones para la Lucha Contra las Enfermedades del Riñón (ALCER), Fundación Renal Íñigo Álvarez de Toledo (FRIAT), Red de Investigación Renal (REDINREN), Resultados en Salud 2040 (RICORS2040), Sociedad Española de Nefrología (SENEFRO) Council, Sociedad Española de Trasplante (SET) Council, Organización Nacional de Trasplantes (ONT) Roger M Jiménez VM Perez JCR Furlano M. RICORS 2040: the need for collaborative research in chronic kidney disease. Clin Kidney J. (2022) 15:372–87. doi: 10.1093/ckj/sfab170, PMID: 35211298 PMC8862113

[4] Paueksakon P Fogo AB. Do proton-pump inhibitors cause CKD and progression of CKD?: COMMENTARY. Kidney. (2022) 3:1141–3. doi: 10.34067/KID.0008302021, PMID: 35920527 PMC9337904

[5] Gherghina M-E Peride I Tiglis M Neagu TP Niculae A Checherita IA. Uric acid and oxidative stress—relationship with cardiovascular, metabolic, and renal impairment. Int J Mol Sci. (2022) 23:3188. doi: 10.3390/ijms23063188, PMID: 35328614 PMC8949471

[6] Mitchell T De Miguel C Gohar EY. Sex differences in redox homeostasis in renal disease. Redox Biol. (2020) 31:101489. doi: 10.1016/j.redox.2020.101489, PMID: 32197946 PMC7212488

[7] León-Aparicio D Salvador C Aparicio-Trejo OE Briones-Herrera A Pedraza-Chaverri J Vaca L. Novel potassium channels in kidney mitochondria: the hyperpolarization-activated and cyclic nucleotide-gated HCN channels. Int J Mol Sci. (2019) 20:4995. doi: 10.3390/ijms20204995, PMID: 31601020 PMC6834191

[8] Krata N Zagożdżon R Foroncewicz B Mucha K. Oxidative stress in kidney diseases: the cause or the consequence? Arch Immunol Ther Exp. (2018) 66:211–20. doi: 10.1007/s00005-017-0496-0, PMID: 29214330 PMC5956016

[9] Tamma G Valenti G. Evaluating the oxidative stress in renal diseases: what is the role for S-Glutathionylation? Antioxid Redox Signal. (2016) 25:147–64. doi: 10.1089/ars.2016.6656 26972776

[10] Lever JM Boddu R George JF Agarwal A. Heme Oxygenase-1 in kidney health and disease. Antioxid Redox Signal. (2016) 25:165–83. doi: 10.1089/ars.2016.6659, PMID: 26906116 PMC4948210

[11] Zwolińska D Grzeszczak W Szczepańska M Kiliś-Pstrusińska K Szprynger K. Vitamins a, E and C as non-enzymatic antioxidants and their relation to lipid peroxidation in children with chronic renal failure. Nephron Clin Pract. (2005) 103:c12–8. doi: 10.1159/000090506, PMID: 16374033

[12] Sahni N Gupta KL. Dietary antioxidents and oxidative stress in predialysis chronic kidney disease patients. J Nephropathol. (2012) 1:134–42. doi: 10.5812/nephropathol.8108, PMID: 24475404 PMC3886144

[13] Locatelli F Canaud B Eckardt KU Stenvinkel P Wanner C Zoccali C. Oxidative stress in end-stage renal disease: an emerging threat to patient outcome. Nephrol Dial Transplant. (2003) 18:1272–80. doi: 10.1093/ndt/gfg074, PMID: 12808161

[14] Hasselwander O Young IS. Oxidative stress in chronic renal failure. Free Radic Res. (1998) 29:1–11. doi: 10.1080/10715769800300011 9733016

[15] Annuk M Zilmer M Lind L Linde T Fellström B. Oxidative stress and endothelial function in chronic renal failure. J Am Soc Nephrol. (2001) 12:2747–52. doi: 10.1681/ASN.V12122747 11729244

[16] Liakopoulos V Roumeliotis S Gorny X Dounousi E Mertens PR. Oxidative stress in hemodialysis patients: a review of the literature. Oxidative Med Cell Longev. (2017) 2017:1–22. doi: 10.1155/2017/3081856, PMID: 29138677 PMC5613374

[17] Ceballos-Picot I Witko-Sarsat V Merad-Boudia M Nguyen AT Thévenin M Jaudon MC. Glutathione antioxidant system as a marker of oxidative stress in chronic renal failure. Free Radic Biol Med. (1996) 21:845–53. doi: 10.1016/0891-5849(96)00233-X, PMID: 8902530

[18] Aveles PR Criminácio CR Gonçalves S Bignelli AT Claro LM Siqueira SS. Association between biomarkers of carbonyl stress with increased systemic inflammatory response in different stages of chronic kidney disease and after renal transplantation. Nephron Clin Pract. (2010) 116:c294–9. doi: 10.1159/000318792, PMID: 20639676

[19] Kuchta A Pacanis A Kortas-Stempak B Çwiklińska A Ziętkiewicz M Renke M. Estimation of oxidative stress markers in chronic kidney disease. Kidney Blood Press Res. (2011) 34:12–9. doi: 10.1159/000321508 21071957

[20] Tejchman K Kotfis K Sieńko J. Biomarkers and mechanisms of oxidative stress—last 20 years of research with an emphasis on kidney damage and renal transplantation. Int J Mol Sci. (2021) 22:8010. doi: 10.3390/ijms22158010, PMID: 34360776 PMC8347360

[21] Van Hoydonck PGA Temme EHM Schouten EG. A dietary oxidative balance score of vitamin C, β-carotene and iron intakes and mortality risk in male smoking Belgians. J Nutr. (2002) 132:756–61. doi: 10.1093/jn/132.4.756, PMID: 11925473

[22] Son D-H Lee HS Seol SY Lee YJ Lee JH. Association between the oxidative balance score and incident chronic kidney disease in adults. Antioxidants. (2023) 12:335. doi: 10.3390/antiox12020335, PMID: 36829895 PMC9952833

[23] Li H Song L Cen M Fu X Gao X Zuo Q. Oxidative balance scores and depressive symptoms: mediating effects of oxidative stress and inflammatory factors. J Affect Disord. (2023) 334:205–12. doi: 10.1016/j.jad.2023.04.134, PMID: 37149058

[24] Zhang W Peng SF Chen L Chen HM Cheng XE Tang YH. Association between the oxidative balance score and telomere length from the National Health and nutrition examination survey 1999-2002. Oxid Med Cell Longev. (2022) 2022:1345071. doi: 10.1155/2022/1345071, PMID: 35186180 PMC8850082

[25] Tian X Xue B Wang B Lei R Shan X Niu J. Physical activity reduces the role of blood cadmium on depression: a cross-sectional analysis with NHANES data. Environ Pollut. (2022) 304:119211. doi: 10.1016/j.envpol.2022.119211, PMID: 35341822

[26] Gor D Gerber BS Walton SM Lee TA Nutescu EA Touchette DR. Antidiabetic drug use trends in patients with type 2 diabetes mellitus and chronic kidney disease: a cross-sectional analysis of the National Health and nutrition examination survey. J Diabetes. (2020) 12:385–95. doi: 10.1111/1753-0407.13003, PMID: 31652390

[27] August P. Chronic kidney disease — Another step forward. N Engl J Med. (2023) 388:179–80. doi: 10.1056/NEJMe2215286 36630627

[28] Levin A Stevens PE. Summary of KDIGO 2012 CKD guideline: behind the scenes, need for guidance, and a framework for moving forward. Kidney Int. (2014) 85:49–61. doi: 10.1038/ki.2013.444, PMID: 24284513

[29] Devraj R Deshpande M. Demographic and health-related predictors of proton pump inhibitor (PPI) use and association with chronic kidney disease (CKD) stage in NHANES population. Res Soc Adm Pharm. (2020) 16:776–82. doi: 10.1016/j.sapharm.2019.08.032, PMID: 31445985

[30] Ilori TO Sun Ro Y Kong SY Gutierrez OM Ojo AO Judd SE. Oxidative balance score and chronic kidney disease. Am J Nephrol. (2015) 42:320–7. doi: 10.1159/000441623, PMID: 26569393 PMC4689189

[31] Nath KA Croatt AJ Hostetter TH. Oxygen consumption and oxidant stress in surviving nephrons. Am J Physiol Renal Physiol. (1990) 258:F1354–62. doi: 10.1152/ajprenal.1990.258.5.F1354, PMID: 2337154

[32] Cross CE Halliwell B Borish ET Pryor WA Ames BN Saul RL. Oxygen radicals and human disease. Davis conference. Ann Intern Med. (1987) 107:526–45. doi: 10.7326/0003-4819-107-4-526 3307585

[33] Ong-Ajyooth L Ong-Ajyooth S Sirisalee K Nilwarangkur S. Lipoproteins and lipid peroxidation abnormalities in patients with chronic renal disease. J Med Assoc Thai. (1996) 79:505–12. 8855633

[34] Yilmaz MI Saglam M Caglar K Cakir E Sonmez A Ozgurtas T. The determinants of endothelial dysfunction in CKD: oxidative stress and asymmetric Dimethylarginine. Am J Kidney Dis. (2006) 47:42–50. doi: 10.1053/j.ajkd.2005.09.029, PMID: 16377384

[35] Himmelfarb J Stenvinkel P Ikizler TA Hakim RM. The elephant in uremia: oxidant stress as a unifying concept of cardiovascular disease in uremia. Kidney Int. (2002) 62:1524–38. doi: 10.1046/j.1523-1755.2002.00600.x, PMID: 12371953

[36] Terawaki H Yoshimura K Hasegawa T Matsuyama Y Negawa T Yamada K. Oxidative stress is enhanced in correlation with renal dysfunction: examination with the redox state of albumin. Kidney Int. (2004) 66:1988–93. doi: 10.1111/j.1523-1755.2004.00969.x, PMID: 15496170

[37] Stringer KD Komers R Osman SA Oyama TT Lindsley JN Anderson S. Gender hormones and the progression of experimental polycystic kidney disease. Kidney Int. (2005) 68:1729–39. doi: 10.1111/j.1523-1755.2005.00589.x 16164649

[38] Carrero JJ Hecking M Chesnaye NC Jager KJ. Sex and gender disparities in the epidemiology and outcomes of chronic kidney disease. Nat Rev Nephrol. (2018) 14:151–64. doi: 10.1038/nrneph.2017.181 29355169

[39] Valdivielso JM Jacobs-Cachá C Soler MJ. Sex hormones and their influence on chronic kidney disease. Curr Opin Nephrol Hypertens. (2019) 28:1–9. doi: 10.1097/MNH.0000000000000463 30320621

[40] Díaz A López-Grueso R Gambini J Monleón D Mas-Bargues C Abdelaziz KM. Sex differences in age-associated type 2 diabetes in rats—role of estrogens and oxidative stress. Oxidative Med Cell Longev. (2019) 2019:1–13. doi: 10.1155/2019/6734836, PMID: 31089412 PMC6476064

[41] Lakkur S Goodman M Bostick RM Citronberg J McClellan W Flanders WD. Oxidative balance score and risk for incident prostate cancer in a prospective U.S. cohort study. Ann Epidemiol. (2014) 24:475–478.e4. doi: 10.1016/j.annepidem.2014.02.015, PMID: 24731700 PMC4352102

[42] Dash C Goodman M Flanders WD Mink PJ McCullough ML Bostick RM. Using pathway-specific comprehensive exposure scores in epidemiology: application to oxidative balance in a pooled case-control study of incident, sporadic colorectal adenomas. Am J Epidemiol. (2013) 178:610–24. doi: 10.1093/aje/kwt007, PMID: 23639935 PMC3816340

